# Small Molecular Contaminant and Microorganism Can Be Simultaneously Detected Based on Nanobody-Phage: Using Carcinogen Aflatoxin and Its Main Fungal *Aspergillus* Section *Flavi* spp. in Stored Maize for Demonstration

**DOI:** 10.3389/fmicb.2019.03023

**Published:** 2020-01-23

**Authors:** Xianfeng Ren, Xiaofeng Yue, Silivano Edson Mwakinyali, Wen Zhang, Qi Zhang, Peiwu Li

**Affiliations:** ^1^Oil Crops Research Institute of the Chinese Academy of Agricultural Sciences, Wuhan, China; ^2^Key Laboratory of Detection for Mycotoxins, Ministry of Agriculture and Rural Affairs, Wuhan, China; ^3^Laboratory of Risk Assessment for Oilseeds Products, Ministry of Agriculture and Rural Affairs, Wuhan, China; ^4^Key Laboratory of Biology and Genetic Improvement of Oil Crops, Ministry of Agriculture and Rural Affairs, Wuhan, China; ^5^Quality Inspection and Test Center for Oilseeds Products, Ministry of Agriculture and Rural Affairs, Wuhan, China

**Keywords:** real-time PCR, aflatoxin, *Aspergillus*, nanobody-phage, *Nor-1* gene

## Abstract

Simultaneous detection technology has become a hot topic in analytical chemistry; however, very few reports on how to simultaneously detect small molecular contaminants and microorganisms have been in place. Aflatoxins are a group of highly toxic and carcinogenic compounds, which are produced mainly by *Aspergillus flavus* and *Aspergillus parasiticus* from section *Flavi* responsible for aflatoxin accumulation in stored cereals. Both aflatoxins and *Aspergillus* section *Flavi* were used to demonstrate the duplex real-time RCR method of simultaneously detecting small molecular contaminants and microorganisms. The detection of aflatoxins and *Aspergillus* section *Flavi* was carried out depending on the anti-idiotypic nanobody-phage V_2–5_ and aflatoxin-synthesis related gene *nor-1* (=*aflD*), respectively. The quantitative standard curves for simultaneous detection of aflatoxins and *Aspergillus* section *Flavi* were constructed, with detection limits of 0.02 ng/ml and 8 × 10^2^ spores/g, respectively. Naturally contaminated maize samples (*n* = 25) were analyzed for a further validation. The results were in good agreement between the new developed method and the referential methods (high-performance liquid chromatography and the conventional plating counts).

## Introduction

Simultaneous detection technology has been becoming a hot topic in analytical chemistry. Many methods have been reported for simultaneous detection of multi small molecular contaminants such as mycotoxins (Li et al., 2013, 2019; Zhang et al., 2016; Wang et al., 2017a), pesticide residues (Bagheri et al., 2016; Wang et al., 2017b), and veterinary drugs (Taranova et al., 2015; Dasenaki et al., 2016; Zhu et al., 2016). Also, a lot of methods were described for simultaneous detection of multi microorganisms such as pathogenic bacteria (Li et al., 2015; Yoo et al., 2015; Vaisocherova-Lisalova et al., 2016), fungal pathogens (Playford et al., 2006; Priyanka et al., 2015; Rahn et al., 2016), and even varied pathogens that belong to different kingdoms (Leber et al., 2016). However, very few reported on how to simultaneously detect small molecular contaminants and microorganisms. In many cases, small molecular contaminants and food-borne microorganisms may simultaneously occur in an identical sample. In this study, we developed a new method for simultaneous detection of aflatoxin and its major fungi in stored maize to demonstrate the potential to simultaneously detect small molecular contaminants and microorganisms.

Aflatoxins are highly toxic, carcinogenic, and mutagenic small molecular contaminants that can not only cause acute or chronic liver diseases but also seriously damage on other tissue organs (Eaton and Gallagher, 1994; Bennett and Klich, 2003). Aflatoxins B_1_, B_2_, G_1_, and G_2_ are the most frequent ones in agricultural products and the most toxic member whereby aflatoxin B1 has been classified as group I human carcinogen by the International Agency for Research on Cancer. In addition, main aflatoxigenic species, namely, *A. flavus* and *A. parasiticus* that belong to *Aspergillus* section *Flavi* (Giorni et al., 2007; Varga et al., 2011) are dominant in infection and colonization of agricultural crops (Desjardins, 2003). *A. flavus* is dominant in invading peanuts, corns, and cottons (Klich, 2007), while *A. parasiticus* contaminates broadly on cereals, oilseeds, spices, and nuts (Reddy et al., 2010). The contaminations triggered by *A. flavus* and *A. parasiticus* result in direct negative effects such as a reduction of production, a loss of nutrition and a diminution of market value, and aggravate environmental especially aqueous pollution and also pose serious threats to the health of animals and humans. The pathogenic *Aspergillus* spp. can cause avian aspergillosis and bovine mycotic abortion, and their spores are associated with human hypersensitivity pneumonitis (Gourama and Bullerman, 1995). Contaminations from aflatoxin and its producing molds usually occur concurrently, which increases a serious dangerousness for people’s health as well as significantly reduces economic values of the host plants, agricultural products, feeds and/or foods.

Currently, a number of quantitative techniques for aflatoxin determination have been developed, mainly including High-Performance Liquid Chromatography (HPLC), Liquid Chromatography tandem Mass Spectrometry (LC-MS), rapid immune-chromatographic assays (ICA) and enzyme-linked immune sorbent assay (ELISA). Methods for quantifying *Aspergillus* section *Flavi* involved morphological and molecular technologies, the former of which need microbiologists who have a rich morphological knowledge to complete, whereas the latter have been widely used because of features of speediness, sensitivity, and accuracy. The present study developed a new method that realized a simultaneous run of two different types of PCR: (1) Display Mediated Immuno-polymerase Chain Reaction (PD-IPCR), which helps to determine total aflatoxins, and (2) a conventional real-time PCR (RT-PCR), which serves for determination of the main aflatoxin-producing fungi *Aspergillus* section *Flavi* in stored maize. Through the combination of the two PCRs, a new detection platform was developed, which makes it possible to simultaneously detect small molecular contaminants and microorganisms.

## Materials and Methods

### Materials

The standard mycotoxin powders, the surfactants Tween-20, and the enzyme stabilizer bovine serum albumin (BSA) were obtained from Sigma-Aldrich (St. Louis, MO, United States). *Escherichia coli* ER2738 competent cells were purchased from Lucigen Corp. (Middleton, WI, United States). The Universal Probes Supermix was supplied by Bio-Rad (Hercules, CA, United States). DNA polymerase (iTaq), Mg^2+^, dNTPs, 6× loading buffer, and DNA marker were bought from Takara Bio (Beijing, China). All the other reagents used were of analytical grade or better.

The anti-aflatoxins monoclonal antibody 1C11 (mAb 1C11) and V_2–5_ phage displaying nanobody specific for 1C11 were produced by our team (Zhang et al., 2009; Wang et al., 2013b). *A. flavus* strain 3.4408 producing a high level of aflatoxins B_1_ and B_2_ was used as a standard strain.

### Preparation of Phage for Small Molecular Contaminant Detection

V_2–5_ phagemids, specific for mAb 1C11, previously transferred to *E. coli* ER2738 and stored at −70°C, need to be released and amplified from the *E. coli*, which was carried out as described in Lei et al. (2014). Finally, the phage particles were titrated by determining phage-forming unit (pfu) and stored at −20°C as ready-to-use reagents to prepare additional supplies if needed.

### Preparation of Reference Gene for Microorganism Detection

*Nor-1* gene, catalyzing the transformation from norsolorinic to averantin, is the first key gene in the pathway of aflatoxins synthesis (Trail et al., 1994; Zhou and Linz, 1999). *A. flavus* strain 3.4408 was used to obtain *nor-1* gene. After the inoculation on Czap ekDox Agar (CDA), the fungus was incubated at 28°C and 90% humidity for 7 days, and then the spores were washed down, counted using a hemocytometer counting chamber, and diluted into 50 ml of potato dextrose broth (PDB) to a final concentration of 1 × 10^5^ spores/ml, followed by a shaking at 180 rpm for 96 h at 28°C using a Thermo Scientific MaxQ 4000 shaker (Danville, CA, United States). Finally, the mycelia were washed three times with double-distilled water, filtered through double-filter papers (Whatman #4, Maidstone, United Kingdom), immediately freeze-dried and stored at −70°C prior to DNA extraction.

DNA was extracted using DNeasy Plant Mini Kit (Qiagen, GmbH, Germany) according to the manufacturer’s introductions. After DNA extraction, a conventional PCR was performed essentially as described by Geisen (1996). The larger fragments (400 bp) of *nor-1* gene were generated with primers: nor1-F, 5′-ACCGCTACGCCGGCACTCTCGGCAC-3′ and nor1-R, 5′-GTTGGCCGCCAGCTTCGACACTCCG-3′. Then, these larger fragments were purified using E.Z.N.A. TM Gel Extraction Kit (Omega Bio-Tek, Norcross, GA, United States) according to the manufacturer’s protocols. Concentration of the purified products was determined by measuring the absorbance of samples at 260 and 280 nm, using a NanoDrop 1000 (Thermo Fisher Scientific, Waltham, MA, United States), and the number of copies was calculated.

### Optimization of the Duplex Real-Time PCR

The primer/probe systems are shown in Table 1. Ph-F, -R primers, and Ph-probe were designed according to the corresponding specific DNA sequences encoding anti-idiotypic nanobody (V_2–5_) (Lei et al., 2014), while Tq-nor1-F, -R primers, and Tq-probe were designed according to the sequences of *nor-1* gene (Mayer et al., 2003a). The probes were labeled with non-fluorescent BHQ1 at the 3′-end and with reporter dyes of FAM or Hex at the 5′-end. Primer Premier 6.0 (Premier Biosoft International, Palo Alto, CA, United States) was used to ensure the compatibility of primers and probes.

**TABLE 1:** Primer and probe systems used in the duplex real-time PCR system.

**Primer/probe**	**Sequence (5′ to 3′)**	***T*_m_ (°C)**	**Amplicon (bp)**	**Working concentration (μM)**	**Target gene**
Ph-F	GTGGTAGCACAAACTATG	49.5	131	0.3	Phage DNA
Ph-R	GGCTGCACAGTAATAAAC	50.2		0.3	
Ph- probe	FAM-CCGATTCACCATCTCCAGAGACA-BHQ1	58.2		0.4	
Tq-nor1-F	GTCCAAGCAACAGGCCAAGT	57.4	66	0.2	*Nor-1* gene
Tq-nor1-R	TCGTGCATGTTGGTGATGGT	55.4		0.2	
Tq-probe	HEX-TGTCTTGATCGGCGCCCG-BHQ1	62.2		0.3	

A CFX96^TM^ real-time PCR system (Bio-Rad, Hercules, CA, United States) was used to perform the real-time PCR assay. The duplex real-time PCR consisted of two single-plex amplification systems that separately used V_2–5_ phage DNA and *nor-1* gene as templates. After the separate optimization, the two single-plex PCRs were combined to form a duplex real-time PCR, with an additional 0.25–1.0 U DNA polymerase (iTaq), Mg^+2^ (1–2 mM), and dNTPs (200–400 μM).

Parameters for the optimized system were as follows: V_2__–__5_ phage (2 μl) and *nor-1* (l μl) were mixed with the PCR working solution containing two primer/probe systems (Table 1), iTaq Universal Probes Supermix (5 μl), an additional iTaq (0.75 U), MgCl_2_ (2 mM), and dNTPs (400 μM). Double-distilled water was added to make up the total volume to 10 μl. After an initial denaturation at 95°C for 5 min, 40 cycles were at 95°C for 10 s and 60°C for 30 s. No template control was used to verify the quality of amplification. All the assays were carried out in triplicate.

To evaluate the amplification efficiency (E), V_2–5_ phage particles were diluted in PBS buffer (10 mM sodium phosphate buffer containing 137 mM NaCl and 2.68 mM KCl, pH 7.4) to a series of final concentrations ranging from 10^9^ to 10^2^ pfu/ml. The reference *nor-1* gene was 10-fold serially diluted in nuclease-free H_2_O to final concentrations of 10^8^–10^1^ copies/μl. Ct values, corresponding to each dilution, were automatically calculated by the instrument. The efficiency was calculated based on: *E* = [10^1/–slope^ – 1] × 100%, by using logarithm of templates as abscissa and Ct values as ordinate to plot amplification calibration curves.

### Immunoreaction for Small Molecular Contaminant Detection

A polystyrene microtiter plate (96-well) was coated with 1.0 μg/ml mAb 1C11 at 37°C for 1 h. Then, the plate was washed with PBST [PBS containing 0.05% (v/v) Tween 20] three times and, then, blocked with a buffer [PBST containing 3% (w/v) skimmed milk] at 37°C for 45 min. The plate was washed three times. Then, the mixture (100 μl) containing 50 μl of V_2__–__5_ phages (1.0 × 10^10^ pfu/ml) and the same volume of aflatoxins solution were added into each micro-plate well. After the incubation at 37°C for 1 h, the plate was washed with PBST 10 times. Subsequently, the V_2__–__5_ phages captured by mAb 1C11 at the bottom of the plate were eluted by Glycine/HCl buffer (100 μl, 0.2 M, pH 2.1, containing 1% BSA) at 37°C for 15 min. Then, the eluent containing the released phages was neutralized using 1 M Tris–base buffer (pH 9.1).

Aflatoxins B_1_, B_2_, G_1_, and G_2_ occur in natural samples at different ratios (Kensler et al., 2011). According to their frequencies of occurrence in natural samples, standards (B_1_: B_2_: G_1_: G_2_ = 1.0: 0.1: 0.3: 0.03, w/w/w/w) used for total aflatoxin determination were prepared. The standard was threefold serially diluted at serial concentrations (33.3 ng/ml to 1.69 pg/ml). After the immunoreaction as described above, the eluted V_2–5_ phages solution (2 μl) was used for the duplex real-time PCR system. The standard curve was constructed by plotting Ct values versus Log total aflatoxin concentrations (Log 10) by four parameter logistic regression.

### Isolation of DNA From Maize Samples

Ten grams of maize was finely milled into particles < 500 μm diameter using a laboratory mill. Subsequently, 0.2 g of the powder was precisely weighed, transferred into a nuclease-free tube, and smashed using an automatic fast-grinding apparatus Tissuelyser-48 (Jingxin Science, Shanghai, China), in the presence of 200 μl of CTAB (hexadecyltrimethyl ammonium bromide) buffer [20 g/L CTAB, 0.1 M Tris–HCl (pH = 8.0), 20 mM EDTA (pH = 8.0) and 1.4 M NaCl] and two steel beads (1.5 mm diameter). Then, an additional 1.6 ml of CTAB buffer was added into the tube and immediately incubated in a water bath at 63°C for 2 h. Then, 1 volume of phenol:chloroform:isoamyl alcohol (v:v:v, 25:24:1) was added, gently mixed, and centrifuged at 15,000 *g* for 10 min. After the recovery of the aqueous phase, 1 volume of chloroform:isoamyl alcohol (v:v = 24:1) was added, homogenized, and centrifuged again. The aqueous phase was recovered and 1 volume of chloroform was added. After the centrifugation at 15,000 *g* for 10 min, the aqueous phase was recovered again, followed by an addition of 0.6 volume of isopropyl alcohol (pre-cooled at −20°C for 2 h). After the centrifugation at 15,000 *g* for 15 min, the aqueous phase was discarded. The DNA was cleaned with 70% alcohol, suspended in 70 μl of nuclease-free H_2_O, and stored at −20°C.

To generate a standard curve for *Aspergillus* section *Flavi* determination, 0.2 g of the finely milled blank-maize powder was precisely weighed, transferred into a nuclease-free tube, and inoculated with 200-μl spores (obtained from 6-day-old *A. flavus* strain 3.4408) to final concentrations of 8 × 10^2^ to 8 × 10^8^ spores/g. After incubation at 28°C for 1 h, samples were used to extract DNA as described above. DNA products (l μl) were used as templates for the duplex real-time PCR system. Log spores/g was used as abscissa and the corresponding Ct values were used as ordinate to plot the standard curve.

### Samples Analysis and Validation

The blank maize samples were purchased at a local market and verified as blank using HPLC and conventional plating counts. The naturally contaminated maize samples (*n* = 25) were gathered from Shandong province of China. Samples (10 g) were finely milled into particles < 500 μm diameter, 0.2 g of which was used to extract DNA for *Aspergillus* section *Flavi* determination. For aflatoxin extraction, 5.0 g of the milled samples was treated with 15 ml of methanol:water (70:30, v/v) under a shaking condition at 250 rpm for 1 h. After a centrifugation (5000 *g* for 10 min at 4°C), samples were filtered with double-filter papers (Whatman #4, Maidstone, United Kingdom) and diluted sevenfold with 4% BSA/PBS (w/v). Dilutions were used directly in the Section “Immunoreaction for Small Molecular Contaminant Detection.” After the immunoreaction and DNA extraction, the eluted phages DNA and DNA products extracted from maize samples were amplified simultaneously in the optimized duplex real-time PCR system. Ct values were associated to standard curves to calculate concentrations of aflatoxins and aflatoxigenic fungi.

A validation involved testing of 25 naturally contaminated maize samples, using the newly developed method and the gold standard reference methods (HPLC and conventional plating counts) in parallel. HPLC was carried out as described in Ren et al. (2019). For determination of *Aspergillus* section *Flavi* density by conventional plating counts, colony-forming units (CFUs) were determined using dichloran rose bengal chloramphenicol agar (DRBC) supplemented with 1% NaCl (Passone et al., 2010).

### Statistical Analysis

For aflatoxin determination, IC_10,_ IC_50_ (half-maximal inhibition), and IC_20_ - IC_80_ were used to calculate the limit of detection (LOD), sensitivity and linear range, respectively. The statistical analysis and plotting were performed using Microsoft Excel 2007 and OriginPro 9.0 (OriginLab Corporation, Northampton, MA, United States). To assess matrix effects, data were compared according to Student *t*-test using Graph PadInstat 3.0 (GraphPad Software, San Diego, CA, United States).

## Results and Discussion

### Optimization of the Duplex Real-Time PCR

**FIGURE 1:**
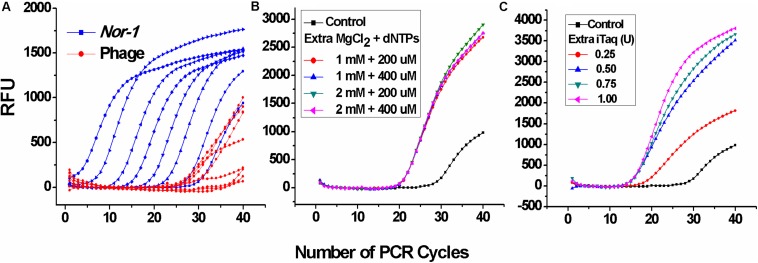
Optimization of iTaq (DNA polymerase), dNTPs, and Mg^2+^ for the duplex real-time PCR. **(A)** Amplification data of the duplex real-time PCR system directly combined by two single-plex systems without any extra reagents. RFU means relative fluorescence units. **(B)** Amplification data of V_2__–__5_ phage at different concentrations of additional MgCl_2_ and dNTPs and **(C)** DNA polymerase (iTaq).

At first, two single-plex real-time PCR systems were directly combined without any further optimization. As shown in Figure 1A, Ct values corresponding to V_2__–__5_ phages were much higher, which indicated that the amplification of phage DNA was extremely inhibited, whereas the additional Mg^+2^, dNTPs (Figure 1B), and DNA polymerase (iTaq) (Figure 1C) enhanced phage DNA amplification with significantly lower Ct values. Principles defining optimal parameters were intended to ensure that positive Ct values are lower, because the lower Ct values ensured a wider linear range and a lower limit of the detection. Thus, the additional 2 mM MgCl_2_, 400 μM dNTPs, and 0.75 U iTaq were selected as the optimal conditions. These results suggest that insufficient DNA polymerase, Mg^+2^, and/or nucleotides can inhibit the amplification of low-abundance templates, which was in accordance with the conclusion of Svec et al. (2015).

### Efficiency Assessment of the Duplex Real-Time PCR

**FIGURE 2:**
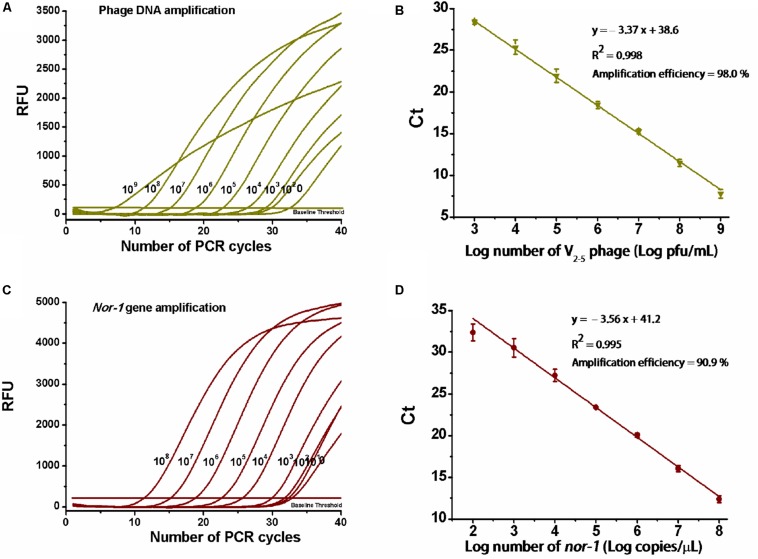
Determination of amplification efficiency of the real-time PCR. **(A)** PCR amplification for 10-fold serial dilutions of V_2__–__5_ phage particles (10^9^,…10^8^,…10^7^,…10^6^,…10^5^,…10^4^,…10^3^,…10^2^ pfu/ml) and **(C)** reference *nor-1* gene (10^8^,…10^7^,…10^6^,…10^5^,…10^4^,…10^3^,…10^2^,…10^1^ copies/μl). RFU means relative fluorescence units. **(B)** Standard curve of amplification efficiency for V_2__–__5_ phage and **(D)** reference *nor-1* gene. Each data point is the average of three independent measurements.

Amplification data are shown in Figures 2A,C. For V_2__–__5_ phage and reference *nor-1* gene, the resulting slopes for linear fit were −3.37 (Figure 2B) and −3.56 (Figure 2D), respectively. Thus, amplification efficiencies were calculated as 98 and 91%, with the lowest detectable concentrations of 10^3^ pfu/ml V_2__–__5_ and 10^2^ copies/μl *nor-1*, indicating that the optimized duplex real-time PCR was accurate enough for simultaneous quantification of the both targets.

### Matrix Effect, Sensitivity, and Specificity for Total Aflatoxin Determination

**FIGURE 3:**
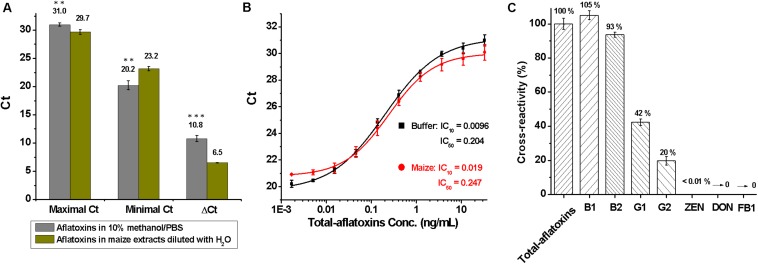
Evaluation of the duplex real-time PCR for total aflatoxins detection. **(A)** Analysis of matrix effects, by comparing the difference of maximal, minimal, and ΔCt values between 10% methanol/PBS buffer and maize extracts that diluted with water; ^∗∗∗^*P* < 0.001, ^∗∗^*P* < 0.01, ^∗^*P* < 0.05 according to the Student *t*-test. **(B)** Standard curves for total aflatoxins in 10% methanol/PBS buffer (black) and maize extracts (red) after the elimination of matrix interference with 4% BSA/PBS (w/v); conc. is the abbreviation for concentration. **(C)** Cross-reactivity (CR) for mycotoxins including total aflatoxins, B_1_, B_2_, G_1_, G_2_, zearalenone (ZEN), deoxynivalenol (DON), and fumonisin B_1_ (FB_1_).

To assess matrix effects on total aflatoxin determination, 5 g of blank maize samples were treated with 15 ml of methanol/PBS (70:30, v/v) under a shaking condition (250 rpm for 1 h), centrifuged at 5000 *g* for 10 min, and filtered through double-filter paper, and then the supernatants were diluted sevenfold with distilled-water. Subsequently, total aflatoxin standard was diluted into 10% methanol/PBS (10:90, v/v) buffer and the dilutions of maize extracts to a final concentration of 33.3 ng/ml to 1.69 pg/ml. Maximal and minimal Ct values were obtained at 33.3 ng/ml and 1.69 pg/ml, respectively. As shown in Figure 3A, ΔCt (=Maximal Ct - Minimal Ct) had a significant difference (*P* < 0.001, according to the Student *t*-test) between 10% methanol/PBS buffer (ΔCt = 10.8) and maize extracts (ΔCt = 6.5), meaning maize matrix had a significant effect on total aflatoxin detection.

To eliminate matrix effects, the maize extracts were diluted sevenfold with 4% BSA/PBS (w/v), which essentially eliminated the matrix interference. Standard curves for total aflatoxins in 10% methanol/PBS buffer and maize extracts that were diluted with BSA/PBS (w/v) are shown in Figure 3B. The LOD, sensitivity, and linear range of the method for total aflatoxins in maize were 0.02, 0.25, and 0.05–1.21 ng/ml, respectively. The LOD was much lower than that of immune-chromatographic assays (Li et al., 2013), immunochip (Wang et al., 2012), and HPLC methods (Khayoon et al., 2010) reported previously.

During assessment of specificity, the cross-reactivity (CR) for common mycotoxins was tested and calculated as: % CR = (IC_50 Total aflatoxins_ /IC_50 analyte_) × 100. As shown in Figure 3C, higher cross-reactivity against total aflatoxins (100%) and aflatoxins B_1_ (105%) and B_2_ (93%), lower cross-reactivity toward aflatoxins G_1_ (42%) and G_2_ (20%), and no cross-reactivity with zearalenone (ZEN), deoxynivalenol (DON), and fumonisin B_1_ (FB_1_) were obtained, indicating that the method was specific for aflatoxins B_1_, B_2_, G_1_, and G_2_.

### Matrix Effect, Sensitivity, and Specificity for *Aspergillus* Section *Flavi* Determination

**FIGURE 4:**
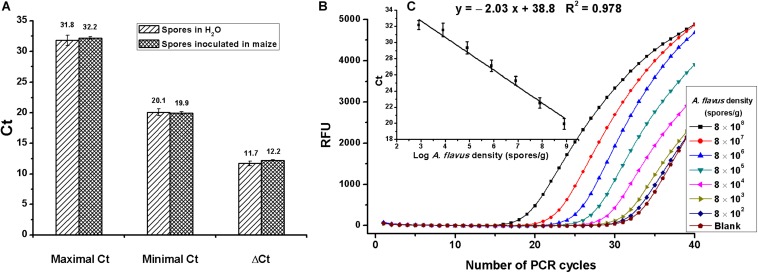
Evaluation of the duplex real-time PCR for *Aspergillus* section *Flavi* determination. **(A)** Analysis of matrix effects, by comparing the difference of maximal, minimal, and ΔCt values between assays that spores diluted in water or inoculated in maize; no difference was found, according to the Student *t*-test. **(B)** Amplification data for serial concentrations of *A. flavus* spores inoculated in maize (8 × 10^8^,…8 × 10^7^,…8 × 10^6^,…8 × 10^5^,…8 × 10^4^,…8 × 10^3^,…8 × 10^2^ spores/g). RFU means relative fluorescence units. **(C)** Standard curves constructed for *Aspergillus* section *Flavi* detection in maize with detection linear range of 8 × 10^2^ to 8 × 10^8^ spores/g.

On assessment of matrix effects on *Aspergillus* section *Flavi* determination, spores were diluted in water or inoculated in maize to serial concentrations of 8 × 10^2^ to 8 × 10^8^ spores/ml or spores/g. As shown in Figure 4A, maximal, minimal, and ΔCt values had no differences between spores inoculated in maize and in water, indicating no matrix effects. Due to the complexity of food samples, food matrices-associated inhibitors such as protein, polysaccharide, and oleic acid usually interfere with the activities of enzymes and, subsequently, reduce the detection sensitivity (Wilson, 1997; Hanna et al., 2005). Fortunately, no matrix inhibition was discovered in this experiment, probably because of the use of phenol and chloroform during the extraction of DNA, which could not only remove proteins but also eliminate other matrix inhibitors.

Amplification data of *A. flavus* spores that were 10-fold serially diluted in maize are shown in Figure 4B. The standard curve for mold detection is shown in Figure 4C. A good linear relationship between Ct values and spore numbers was obtained, with detective standard curve: *y* = −2.03*x* + 38.8 and *R*^2^ = 0.98. As shown in Figures 4B,C, the linear range for *A. flavus* detection was 8 × 10^2^ to 8 × 10^8^ spores/g, with the lowest detectable concentration of 8 × 10^2^ spores/g.

The specificity of the primer/probe set of *nor-1* has been already demonstrated, using the purified genomic DNA of different food-related fungi (Mayer et al., 2003a; Iheanacho et al., 2014). Their studies showed that *A. flavus* and *A. parasiticus* gave positive results, whereas other tested strains such as different *Aspergillus* spp., *Penicillium* spp., and *Fusarium* spp. gave negative results. In our study, the specificity was also tested using strains commonly occurred in maize. As expected, *A. parasiticus* (*n* = 3) and *A. flavus* (*n* = 4, including two aflatoxin non-producing strains) gave PCR amplifications similar to that of *A. flavus* strain 3.4408 (data not shown), indicating that the new method could detect *A. parasiticus* and *A. flavus* including aflatoxin producing and non-producing strains, whereas no PCR amplifications were detected for the other tested strains (*Aspergillus niger*, *Aspergillus nidulans, Penicillium oxalicum*, *Fusarium moniliforme*, *Fusarium nivale*, *Alternaria alternate*, *Trichoderma harzianum*, and *Rhizopus nigricans*).

Regarding *nor-1* gene as a biomarker for *A. flavus* and *A. parasiticus* detection has been demonstrated for several times. Mayer et al. (2003b) demonstrated that the tendency of *nor-1* gene copies was the same as that of *A. flavus* CFUs in wheat with the prolonged incubation time (Mayer et al., 2003b). Additionally, *nor-1* copies were demonstrated to be correlated to CFUs of *A. flavus* in pepper, maize, and paprika (Bagnara et al., 2000; Mayer et al., 2003a). Passone et al. (2010) also developed an analytical method determining *Aspergillus* section *Flavi* based on *nor-1* gene and demonstrated a good correlation (*r* = 0.613; *P* < 0.0001) between *nor-1* copies and CFUs in naturally stored peanut. These results indicated that the PCR system based on *nor-1* gene was specific and accurate for *A. flavus* and *A. parasiticus* determination, which was in accordance with our finding.

At present, some other methods based on PCRs have also been established to detect aflatoxigenic fungi in agricultural products. For example, an analytical method determining CFU values of *Aspergillus* section *Flavi* in stored peanut samples was established, with a detection linear range of 2.5 × 10^3^ to 10^7^cfu/g (Passone et al., 2010), a lower sensitivity compared with that of our method. The method based on omt-1 gene was also proposed to quantify aflatoxin-producing molds, over the range 4 to 1 log cfu/g (Rodriguez et al., 2012). Since mycelial fragments consist of many multinucleate cells (Jennings and Lysek, 1996; Kaminskyj and Hamer, 1998), but give only one colony in a plate, CFU values could not mirror the real density of *Aspergillus* section *Flavi* in samples. Thus, our new method, based on the detection of spores, was more sensitive and accurate.

### Recovery of Total Aflatoxins and *A. flavus* Spores

To test the recovery, blank maize samples (10 g) were spiked with total aflatoxin standard (10, 100, and 200 μg/kg) and simultaneously inoculated with fresh spores of *A. flavus* 3.4408 (3, 5, and 8 Log spores/g). Assays were carried out in triplicate on the same day for intra-assay precision evaluation and in five different days for inter-assay precision evaluation. Recoveries for aflatoxins and *A. flavus* spores were 84–111% and 94–107%, respectively, with variable coefficients (CVs) of 0.47–11.2% (Table 2), indicating a good repeatability and reproducibility of the method.

**TABLE 2:** Recovery of total aflatoxins and *A. flavus* in maize by the duplex real-time PCR analysis.

**Assay**	**Analyte**	**Spiked**	**Recovered**	**Recovery**	**CV**
		**level**	** level ± SD**	**(%)**	**(%)**
Intra-assay	Total aflatoxins	10	8.84 ± 0.30	88.4	3.43
(*n* = 3)^a^	(μg/kg)	100	92.1 ± 6.12	92.1	6.65
		200	206 ± 5.50	103	2.67
	*A. flavus*	3	2.83 ± 0.24	94.4	8.59
	(log spores/g)	5	5.10 ± 0.36	102	6.70
		8	8.57 ± 0.37	107	4.29
Interassay	Total aflatoxins	10	8.39 ± 0.04	83.9	0.47
(*n* = 5)^b^	(μg/kg)	100	43.9 ± 1.29	87.8	2.94
		200	111 ± 3.57	111	3.23
	*A. flavus*	3	3.04 ± 0.34	101	11.2
	(log spores/g)	5	4.89 ± 0.30	97.8	6.13
		8	8.15 ± 0.38	102	4.60

*^*a*^Each assay was carried out in triplicate on the same day. ^*b*^The interassay was carried out in five different days.*

### Validation

**FIGURE 5:**
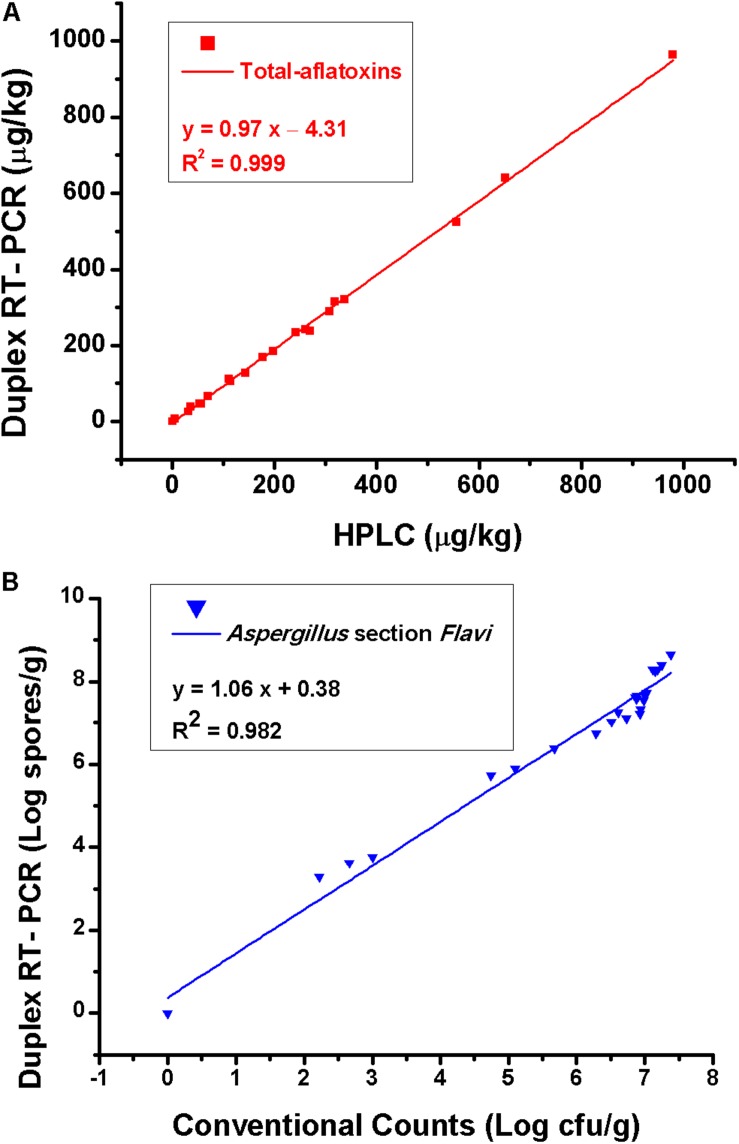
**(A)** Correlation of total aflatoxins results obtained by the duplex real-time PCR and HPLC and **(B)** results of *Aspergillus* section *Flavi* obtained by the duplex real-time PCR and conventional plating counts. Each data point is the average of five independent measurements.

The testing results of 25 natural samples and correlations of the results obtained by different methods are shown in Table 3 and Figure 5. For total aflatoxin determination, results of the new method and HPLC had a good correlation, with a linear regression equation: *y* = 0.97*x* – 4.31 and *R*^2^ = 0.99; for *Aspergillus* section *Flavi*, validation results were also in good agreement, with a linear regression equation: *y* = 1.06*x* + 0.38 and *R*^2^ = 0.98 (Figure 5).

**TABLE 3:** Comparison of results obtained by the duplex real-time PCR and referential methods for total aflatoxins and *Aspergillus* section *Flavi* detection in naturally contaminated maize.

**Maize**	**Total aflatoxins**		***Aspergillus* section**	
**sample**	**concentration**		***Flavi* density**	
	**HPLC**	**Duplex RT-PCR**	**Plating counts**	**Duplex RT-PCR**
	**(ng/ml)**	**(ng/ml)**	**(Log cfu/g)**	**(Log spores/g)**
1	^a^ND	0.53	ND	ND
2	ND	ND	2.67	3.63
3	ND	ND	ND	ND
4	ND	ND	2.23	3.29
5	ND	0.45	3.00	3.76
6	114	106	6.28	6.75
7	53.6	46.2	6.94	7.33
8	198	185	6.98	7.55
9	111	110	7.01	7.66
10	32.0	25.9	6.86	7.61
11	57.2	47.4	6.88	7.66
12	70.7	66.7	6.88	7.58
13	177	169	7.12	8.29
14	143	127	7.15	8.25
15	651	640	7.19	8.28
16	241	233	6.51	7.03
17	269	237	6.73	7.10
18	261	241	7.02	7.73
19	308	288	6.93	7.22
20	337	321	7.38	8.65
21	318	314	6.61	7.25
22	5.19	6.13	4.74	5.74
23	36.2	38.2	5.10	5.90
24	556	524	5.67	6.39
25	980	965	7.25	8.40

*^*a*^ND, not detectable. All assays were carried out in five replicates.*

### Application Prospect

According to the sample analysis protocol, aflatoxins in maize samples were 21-fold diluted, meaning the LOD, sensitivity, and linear range for total aflatoxin detection in maize were 0.42, 5.25, and 1.05–25.41 μg/kg, respectively, and linear range for *Aspergillus* section *Flavi* detection was 8 × 10^2^ to 8 × 10^8^ spores/g. Additionally, approximately 2 h was needed for samples preparation, 4 h for the Section “Immunoreaction for Small Molecular Contaminant Detection,” 4 h for the Section “Isolation of DNA from Maize Samples,” and 1 h for the analysis using real-time PCR instrument. Therefore, approximately 11 h was enough for the whole detection period.

With all of the above performance, this newly developed method was a good demonstration for simultaneous detection of small molecular contaminants and microorganisms in agro-foods. Generally, if nanobody phages specific for small molecular contaminants are available, the simultaneous detection would become not a challenge. Currently, nanobody phages specific for various contaminants such as zearalenone (Wang et al., 2016), ochratoxin A (Liu et al., 2014), deoxynivalenol (Tu et al., 2012), fumonisin B_1_ (Shu et al., 2019), synthetic micro-organics (Wang et al., 2013a; Hua et al., 2015; Ding et al., 2017), citrinin (CIT) (Xu et al., 2015), and microcystins (MCs) (Xu et al., 2018) are available. Therefore, using the new method developed here, the simultaneous detection for these small molecular contaminants and their related microorganisms could also be realized.

## Conclusion

In order to provide an analytical technology to detect small molecular contaminants and microorganisms, the simultaneous detection of aflatoxins and its major fungi (*Aspergillus flavus* and *A. parasiticus*) in maize was developed as an example to demonstrate it. The entire process for the simultaneous detection requires less than 1 day, thus time saving compared with separate detections. Importantly, this technical platform not only achieved the goal of simultaneous quantifications but also satisfied technical features of high throughput, high sensitivity, and wider linear range. However, the tedious technical procedure might be considered as inefficiency at current stage, especially on the procedure of the DNA isolation. Therefore, simplifying protocols for samples preparation are necessary to be explored, further to save time and improve work efficiency. Overall, this detection platform had a great potential for simultaneous detection of small molecular contaminants and microorganisms, which could, in a significant measure, advance new ideas for the development of detection technologies.

## Data Availability Statement

The datasets generated for this study are available on request to the corresponding author.

## Author Contributions

PL and QZ conceived the research and acquired the funding. QZ, PL, and XR designed the experiments and analyzed the data. The manuscript was written through contributions of all authors. All authors have given approval to the final version of the manuscript.

## Conflict of Interest

The authors declare that the research was conducted in the absence of any commercial or financial relationships that could be construed as a potential conflict of interest.
